# The association between health literacy and screening for disease-specific complications among community-dwelling adults with diabetes

**DOI:** 10.3389/fpubh.2024.1418828

**Published:** 2024-09-04

**Authors:** Fatima Nari, Jae Kwan Jun, Kyoung Hee Oh, Wonjeong Jeong

**Affiliations:** ^1^Division of Cancer Early Detection, National Cancer Control Institute, National Cancer Center, Goyang, Republic of Korea; ^2^Cancer Knowledge & Information Center, National Cancer Control Institute, National Cancer Center, Goyang, Republic of Korea

**Keywords:** health literacy, diabetes complications screening, diabetic nephropathy, diabetic retinopathy, diabetes complication disease

## Abstract

**Introduction:**

Diabetic retinopathy and nephropathy are examples of complications of uncontrolled diabetes. We hypothesized that health literacy has a defining role in understanding the importance of attending routine screening for diabetes complications. Therefore, our study investigated the relationship between verbal health literacy (VHL) and written health literacy (WHL) and screening for disease-specific complications in individuals with diabetes.

**Methods:**

Cross-sectional data on 28,210 participants with diabetes was derived from the 2021 Korean Community Health Survey. Adjusted multiple logistic regression analysis was employed to investigate the association between VHL and WHL and diabetes complication screening. Further analysis was also carried out to further comprehend the relationship between those two forms of health literacy and other factors with diabetic retinopathy and nephropathy screening.

**Results:**

Compared to those with high VHL, participants with low VHL had lower odds of diabetes complication screening; OR 0.89 (95% CI 0.84—0.95). The same was true for WHL, those who were uninterested reported the lowest odds ratio; OR 0.73 (95% CI 0.69—0.78), followed by low WHL; OR 0.88 (95% CI 0.82—0.94), of undergoing diabetes complication screening, when compared to individuals with high WHL. Our subgroup analysis presented similar results for diabetic nephropathy and retinopathy with both VHL and WHL.

**Conclusion:**

Among individuals with diabetes, limited VHL and WHL was significantly associated with lower odds of diabetes complication screening. Interventions aimed at improving health literacy and associated health outcomes in the community setting are warranted.

## Introduction

Health literacy is defined as “the extent to which individuals can obtain, process and understand basic health information and services and needed to make appropriate health decisions” (1, 2). This concept encompasses not only the ability to comprehend and assimilate verbal instruction from health care professionals and written health information such as those listed online and medication leaflets, but also other relevant concepts such as understanding health numeracy and implication in cultural context (3). Low health literacy has been linked to adverse health behaviors and outcomes, particularly among chronic disease patients where self-management is crucial to avoid hospitalization, increased health care costs and mortality (4–6).

Diabetes stands as one of the most prevalent chronic diseases on a global scale and in Korea (7, 8). Uncontrolled diabetes may lead to life-threatening microvascular complications such as diabetic nephropathy and retinopathy which may in turn, cause irreversible end stage renal disease and blindness, respectively (9–11). Therefore, timely diagnosis and management including regular screening are necessary to prevent disease-specific complications in patients with diabetes (12). It was previously estimated that the screening rate for diabetic microvascular complications such as diabetic nephropathy and retinopathy was only about 37% in a sample of community dwelling Korean adults with diabetes (13).

Additionally, high rates of insufficient health literacy were reported to be present among individuals with diabetes (2). Limited health literacy may serve as a barrier to understanding instructions to self-management including screening attendance, glycemic control and overall diabetes progression prevention (14, 15). In relation to this, there has been published research in the Korean setting regarding health literacy and chronic disease management of diabetes. For example, a cross-sectional study by Kim et al. (16) found a significant correlation between electronic health literacy and health seeking behaviors in Korean diabetes patients. However to date, studies on health literacy and diabetes-specific complication screening are relatively scarce. Prior evidence suggests that diabetic nephropathy and retinopathy is one of the most common complications in adults with diabetes, stipulating the need to investigate screening behaviors in this at-risk population (17). Adequate health literacy is essential for these individuals to make informed health decisions including seeking preventative screening services (18). Therefore, the aim of our study was to investigate the association between level of verbal health literacy (VHL) and written health literacy (WHL) and diabetic screening complications using a nationally representative sample of community-dwelling South Korean adults with diabetes.

## Methods

### Participants

Cross-sectional data for this study was taken from a sample of 2021 Korean Community Health Survey (KCHS) data, which is a nationwide health interview survey carried out by the Korean Centers for Disease Control and Prevention (KCDC) (19). The KCHS has been conducted to support future health-related policies by understanding of the current health status and aforementioned key characteristics of the population which includes a large portion of the population and contains basic questions regarding sociodemographic and economic factors (20). We extracted individuals who were diagnosed with diabetes. Of these participants, individuals over the age of 20 years were included in this study. Individuals with missing variables, such as household income level, region, diagnosis of hypertension were excluded. Subsequently, a total of 28,210 individuals were included in this study.

### Variables

Diabetic complication screening, the outcome variable in this study, was defined as having underwent screening for diabetic retinopathy or nephropathy. Diabetic complication screening was additionally analyzed by dividing into diabetic retinopathy screening and diabetic nephropathy screening. Screening for diabetic retinopathy and nephropathy was based on the following questions: “Have you ever had an eye examination to see if diabetic eye complications occurred during the past year?” and “Have you ever had a precise urine test (microalbuminuria) to see if diabetic complications in the kidneys developed during the past year (with the exception of the stick test)?” (21).

Both VHL and WHL were evaluated in this study. Regarding VHL, we accordingly classified the variable into high literacy and low literacy based on the ability to comprehend verbal health information such as verbal explanations by clinicians. WHL was defined in our study as the ability to understand written health information such as the internet or brochure, we classified our variable as high literacy and low literacy, as well as uninterested, which referred to those who answered that they do not prefer written health information (22).

We controlled for covariates such as age, sex, marital status, household income level, region, alcohol status, smoking status, self-reported health status, and diagnosis of hypertension.

### Statistical analysis

A chi-square test was conducted to investigate the general characteristics of the study population. Multiple logistic regression analysis was performed to examine the associations of health literacy level and diabetes complication screening, after adjusting for potential confounding variables. Results were reported as odds ratio (OR) and confidence interval (CI). Differences were considered statistically significant with a *p*-value <0.05. All data analyses used SAS 9.4 software.

## Results

General characteristics of our study participants are delineated in Table 1. Among 28,210 participants, 50.2% of individuals with low VHL reported to have undergone diabetic complication screen as opposed to 57.0% of those with high VHL. Additionally, in terms of WHL, 47.3% of those who were reported to be uninterested and 53.0% of those who had low WHL vs. 59.5% of those who had high WHL reported to have screened for diabetes complications.

**Table 1:** General characteristics of study participants.

Variables		Total		Diabetes complication screening				*p*-value
				Yes		No		
		*N*	(%)	*N*	(%)	*N*	(%)	
Verbal Health Literacy (VHL)								<0.0001
High		19,848	(70.4)	11,305	(57.0)	8,543	(43.0)	
Low		8,362	(29.6)	4,195	(50.2)	4,167	(49.8)	
Written Health Literacy (WHL)								<0.0001
High		14,371	(50.9)	8,556	(59.5)	5,815	(40.5)	
Low		6,966	(24.7)	3,694	(53.0)	3,272	(47.0)	
Uninterested		6,873	(24.4)	3,250	(47.3)	3,623	(52.7)	
Age (years)								<0.0001
19–39		592	(2.1)	310	(52.4)	282	(47.6)	
40–49		1,667	(5.9)	967	(58.0)	700	(42.0)	
50–59		4,608	(16.3)	2,724	(59.1)	1,884	(40.9)	
60–69		8,831	(31.3)	5,237	(59.3)	3,594	(40.7)	
70–79		8,523	(30.2)	4,522	(53.1)	4,001	(46.9)	
≥ 80		3,989	(14.1)	1,740	(43.6)	2,249	(56.4)	
Sex								<0.0001
Male		14,166	(50.2)	7,947	(56.1)	6,219	(43.9)	
Female		14,044	(49.8)	7,553	(53.8)	6,491	(46.2)	
Marital status								<0.0001
Married		18,655	(66.1)	10,650	(57.1)	8,005	(42.9)	
Separated or divorced		8,500	(30.1)	4,253	(50.0)	4,247	(50.0)	
Unmarried		1,055	(3.7)	597	(56.6)	458	(43.4)	
Household income level								<0.0001
Low		7,545	(26.7)	3,586	(47.5)	3,959	(52.5)	
Middle		14,090	(49.9)	7,870	(55.9)	6,220	(44.1)	
High		6,575	(23.3)	4,044	(61.5)	2,531	(38.5)	
Region								<0.0001
Metropolitan		6,522	(23.1)	4,242	(65.0)	2,280	(35.0)	
City		5,004	(17.7)	2,924	(58.4)	2,080	(41.6)	
Rural		16,684	(59.1)	8,334	(50.0)	8,350	(50.0)	
Alcohol status								<0.0001
Never		8,605	(30.5)	4,537	(52.7)	4,068	(47.3)	
Ever		19,605	(69.5)	10,963	(55.9)	8,642	(44.1)	
Smoking status								0.1871
Never		16,388	(58.1)	8,950	(54.6)	7,438	(45.4)	
Ever		11,822	(41.9)	6,550	(55.4)	5,272	(44.6)	
Self-reported health status								0.0006
High		5,629	(20.0)	2,964	(52.7)	2,665	(47.3)	
Middle		12,410	(44.0)	6,895	(55.6)	5,515	(44.4)	
Low		10,171	(36.1)	5,641	(55.5)	4,530	(44.5)	
Diagnosis of hypertension								0.0058
Yes		17,441	(61.8)	9,471	(54.3)	7,970	(45.7)	
No		10,769	(38.2)	6,029	(56.0)	4,740	(44.0)	
Total		28,210	(100.0)	15,500	(54.9)	12,710	(45.1)	

Table 2 presents the logistic regression results of VHL and WHL and diabetes complication screening. Compared to those with high VHL, participants with low VHL had lower odds of diabetes complication screening; OR 0.89 (95% CI 0.84—0.95). Regarding WHL, those who were uninterested reported the lowest odds ratio; OR 0.73 (95% CI 0.69—0.78), followed by low WHL; OR 0.88 (95% CI 0.82—0.94), of undergoing diabetes complication screening, when compared to individuals with high WHL.

**Table 2:** Factors associated with screening for diabetes complication.

Variables	Diabetes Complication Screening	
	Adjusted OR	95% CI
Verbal Health Literacy (VHL)		
High	1.00	
Low	0.89	(0.84–0.95)
Written Health Literacy (WHL)		
High	1.00	
Low	0.88	(0.82–0.94)
Uninterested	0.73	(0.69–0.78)
Age (years)		
19–39	0.75	(0.62–0.91)
40–49	1.00	
50–59	1.12	(1.00–1.26)
60–69	1.27	(1.14–1.42)
70–79	1.07	(0.95–1.20)
≥ 80	0.80	(0.70–0.91)
Sex		
Male	1.05	(0.97–1.13)
Female	1.00	
Marital status		
Married	1.00	
Separated or divorced	0.90	(0.85–0.95)
Unmarried	1.05	(0.91–1.21)
Household income level		
Low	0.74	(0.68–0.80)
Middle	0.87	(0.81–0.92)
High	1.00	
Region		
Metropolitan	1.00	
City	0.76	(0.70–0.82)
Rural	0.56	(0.53–0.60)
Alcohol status		
Never	1.00	
Ever	1.03	(0.98–1.10)
Smoking status		
Never	1.00	
Ever	0.89	(0.82–0.96)
Self-reported health status		
High	1.00	
Middle	1.18	(1.10–1.26)
Low	1.42	(1.33–1.53)
Diagnosis of hypertension		
Yes	0.99	(0.94–1.05)
No	1.00	

Subgroup analyses results of factors associated with diabetic nephropathy screening and diabetic retinopathy screening are shown in Table 3. Participants who had low VHL had lower OR for diabetic retinopathy screening; OR 0.90 (95% CI 0.84—0.95) and diabetic nephropathy screening; 0.88 (95% CI 0.82—0.93) compared to their counterparts. For WHL, individuals who were uninterested or who had low levels of WHL showed lower odds ratio of diabetic retinopathy screening; OR 0.76 (95% CI 0.71—0.81) and OR 0.86 (95% CI 0.81—0.93), respectively, when compared to individuals with high WHL. Similarly, those who were uninterested or who had low WHL had lower odds of diabetic nephropathy screening; OR 0.75 (95% CI 0.70—0.80) and OR 0.90 (95% CI 0.84—0.96), as opposed to their counterparts. Furthermore, other associated factors with diabetic retinopathy and nephropathy screening in Table 3 included; those in their 60s; OR 1.49 (95% CI 1.33—1.67), OR 1.15 (95% CI 1.03—1.29), as well as low; OR 1.41 (95% CI 1.31—1.52), OR 1.34 (95% CI 1.25—1.44) and middle self-reported health status; OR 1.14 (95% CI 1.07—1.22),1.16 (95% CI 1.09—1.24) had higher odds of diabetic retinopathy screening and diabetic nephropathy screening, respectively, compared to their counterparts.

**Table 3:** Factors associated with screening for diabetic retinopathy and nephropathy.

Variables	Diabetes complication screening					
	Diabetic retinopathy screening		Diabetic nephropathy screening			
	Adjusted OR	95% CI	Adjusted OR	95% CI		
Verbal Health Literacy (VHL)						
High	1.00		1.00			
Low	0.90	(0.84–0.95)	0.88	(0.82–0.93)		
Written Health Literacy (WHL)						
High	1.00		1.00			
Low	0.86	(0.81–0.93)	0.90	(0.84–0.96)		
Uninterested	0.76	(0.71–0.81)	0.75	(0.70–0.80)		
Age (years)						
19–39	0.85	(0.69–1.04)	0.80	(0.66–0.97)		
40–49	1.00		1.00			
50–59	1.28	(1.14–1.44)	1.04	(0.92–1.16)		
60–69	1.49	(1.33–1.67)	1.15	(1.03–1.29)		
70–79	1.36	(1.21–1.54)	1.00	(0.89–1.13)		
≥ 80	1.04	(0.91–1.19)	0.77	(0.68–0.88)		
Sex						
Male	1.04	(0.96–1.12)	1.08	(1.00–1.16)		
Female	1.00		1.00			
Marital status						
Married	1.00		1.00			
Separated or divorced	0.90	(0.85–0.95)	0.91	(0.85–0.96)		
Unmarried	0.97	(0.84–1.11)	0.99	(0.86–1.14)		
Household income level						
Low	0.73	(0.68–0.80)	0.72	(0.67–0.78)		
Middle	0.89	(0.83–0.95)	0.87	(0.82–0.93)		
High	1.00		1.00			
Region						
Metropolitan	1.00		1.00			
City	0.79	(0.73–0.85)	0.72	(0.67–0.77)		
Rural	0.62	(0.59–0.66)	0.54	(0.51–0.57)		
Alcohol status						
Never	1.00		1.00			
Ever	0.96	(0.91–1.02)	0.99	(0.94–1.05)		
Smoking status						
Never	1.00		1.00			
Ever	0.85	(0.79–0.92)	0.93	(0.86–1.00)		
Self-reported health status						
High	1.00		1.00			
Middle	1.14	(1.07–1.22)	1.16	(1.09–1.24)		
Low	1.41	(1.31–1.52)	1.34	(1.25–1.44)		
Diagnosis of hypertension						
Yes	0.94	(0.90–0.99)	1.05	(1.00–1.10)		
No	1.00		1.00			

## Discussion

Low VHL was associated with 11% lower odds of diabetes complication screening, and low or disinterest in WHL was associated with 12 and 27% lower odds of having underwent diabetes complication screening in our present study. These results held true even when we further examined the relationship between VHL and WHL and diabetic retinopathy and diabetic nephropathy screening separately. The proposed framework by which health literacy may affect health outcomes in diabetic individuals, including screening for diabetes complications, entails an interplay between a plethora of factors, e.g., prior knowledge of health and health behaviors, reading and verbal fluency, and complexity of written and spoken messages (23, 24). VHL may affect the relationship between patient and healthcare provider and resulting chronic disease health outcomes because it plays a significant role in the patient’s ability to comprehend physician’s instructions regarding treatment plans and communicate their own decision-making process (25, 26). Similarly, low levels or disinterest in WHL may be implicated in poor compliance to diabetes complication screening guidelines since majority of health information and education material are provided in print form (27).

There is a paucity of studies on health literacy and diabetes complication screening, making comparison to prior literature difficult. Nevertheless, one cross-sectional study explored the influence of health literacy on several processes of care among adults with diabetes, including having undergone a urine test for microalbuminuria or eye examination by healthcare professionals. Contrary to our present study’s results, there were no significant associations between limited health literacy and the aforementioned indicators (28). On the other hand, our findings were consistent with a recent study using KCHS data, which investigated the effect of verbal and written health literacy on health behaviors in a sample of Korean adults. Individuals who had difficulty in understanding or showed disinterest in health literacy were less likely to engage in preventative health behaviors such as attending medical screening in the past 2 years (22).

Sociodemographic factors such as education and income are known to influence health literacy and healthcare utilization while also simultaneously being independent from it (1, 29). In our study, social disadvantage arising from factors such as older age, unmarried and separated or divorced status, lower household income level and living in non-metropolitan region were significantly associated with decreased odds of receiving both diabetic retinopathy and nephropathy screening in diabetes patients. Several studies cited finances as a major barrier to diabetes complication screening, which is in line with our study’s finding (30, 31). Screening for microvascular complications such as diabetic retinopathy and nephropathy is often costly, dissuading low-income and underinsured individuals from seeking timely care (32). Another finding of note is the reduced odds of diabetes complication screening in rural areas and smaller cities as opposed to metropolitan areas. Proposed potential barriers to diabetes complication screening in rural regions may include lack of accessibility to screening facilities and transportation services, as well as decreased quality of care (30, 33). Local governments must put forth efforts to mitigate these aforementioned inequities, particularly in under deserved areas, by prioritizing and allocating healthcare resources to optimize diabetes outcomes.

Our results are also further corroborated by a study by Lee et al. (34) who reported socioeconomic inequalities in diabetic retinopathy and nephropathy screening uptake in community-dwelling Korean adults with diabetes, irrespective of whether they had received education on diabetes. While the authors did not take into account the study participants’ health literacy level, results suggested that diabetes education alone could not overcome socioeconomic barriers to screening uptake (34). We believe that education programs specifically targeted at improving health literacy, particularly among socioeconomically disadvantaged strata, may benefit diabetic individuals and help them make informed health decisions, which may subsequently increase screening for diabetes-related complications.

Policy implications of the present study results underscore the need to establish a robust and comprehensive health literacy evaluation tool in place for use nationwide across Korea (35). Countries such as the US have assessed health literacy on the national level, and this in turn has aided in shaping policies and strategies to address health literacy related concerns (18, 36). By tackling the general Korean population’s health literacy needs, healthcare costs associated with diabetic microvascular complications and burden on the healthcare system can subsequently be reduced as well.

The main strength of our study is that to our knowledge, our study is the first to investigate the influence of health literacy on screening for diabetes related complications in a large scale survey of Korean adults. Additionally, the use of a large data sample that is nationally representative of the population was ensured by multistage stratified sampling from all regions in Korea. Despite this, there are several limitations of note. First, health literacy in our study was measured using a simple question for VHL and WHL, and not through the use of an extensive health literacy scale or questionnaire (37). Second, the present study design was of cross-sectional nature, ruling out the possibility of causal inference and therefore must be interpreted with caution. Longitudinal studies are required to further comprehend this association. Lastly, although national representativeness was ensured in our study sample, this is generalizable solely to the mostly homogenous Korean population and not to other populations.

## Conclusion

Low VHL and WHL was significantly associated with decreased odds of diabetes complication screening. Further studies are necessary to address the gap in literature between health literacy and screening for diabetes complications. Healthcare professionals should be encouraged to provide assistance to and enhance information exchange and communication with patients with inadequate health literacy. From a policy perspective, provision of tailored programs aimed at improving health literacy is essential to lower diabetes-and related complications-specific burden on the healthcare system.

## Data Availability

Publicly available datasets were analyzed in this study. This data can be found here: The raw data from KCHS were downloaded from the KCHS website (https://chs.kdca.go.kr/chs) after obtaining permission from the administrator.
